# Conversion of 3-amino-4-aryl­amino-1*H*-iso­chromen-1-ones to 1-aryl­iso­chromeno[3,4-*d*][1,2,3]triazol-5(1*H*)-ones: synthesis, spectroscopic characterization and the structures of four products and one ring-opened derivative

**DOI:** 10.1107/S2053229620003757

**Published:** 2020-04-20

**Authors:** Daniel E. Vicentes, Andrea L. Romero, Ricuarte Rodríguez, Justo Cobo, Christopher Glidewell

**Affiliations:** a Facultad de Ciencias, Universidad de Ciencias Aplicadas y Ambientales, Calle 222, No. 55-37, Bogotá, Colombia; b Departamento de Química, Universidad Nacional de Colombia, Cuidad Universitaria, Carrera 30, No. 45-03, Edificio 451, Bogotá, Colombia; c Departamento de Química Inorgánica y Orgánica, Universidad de Jaén, 23071 Jaén, Spain; d School of Chemistry, University of St Andrews, Fife KY16 9ST, Scotland

**Keywords:** synthesis, heterocyclic com­pounds, isocoumarins, triazoles, crystal structure, mol­ecular conformation, hydrogen bonding, supra­molecular assembly

## Abstract

An efficient synthesis of 1-aryl­isochromeno[3,4-*d*][1,2,3]triazol-5(1*H*)-ones, involving the diazo­tization of 3-amino-4-aryl­amino-1*H*-isochromen-1-ones in weakly acidic solution, has been developed and the spectroscopic characterization and crystal structures of four examples are reported. The mol­ecules of 1-phenyl­isochromeno[3,4-*d*][1,2,3]triazol-5(1*H*)-one, C_15_H_9_N_3_O_2_, (I), are linked into sheets by a combination of C—H⋯N and C—H⋯O hydrogen bonds, while the structures of 1-(2-methyl­phen­yl)isochromeno[3,4-*d*][1,2,3]triazol-5(1*H*)-one, C_16_H_11_N_3_O_2_, (II), and 1-(3-chloro­phen­yl)isochromeno[3,4-*d*][1,2,3]triazol-5(1*H*)-one, C_15_H_8_ClN_3_O_2_, (III), each contain just one hydrogen bond which links the mol­ecules into simple chains, which are further linked into sheets by π-stacking inter­actions in (II) but not in (III). In the structure of 1-(4-chloro­phen­yl)isochromeno[3,4-*d*][1,2,3]triazol-5(1*H*)-one, (IV), isomeric with (III), a combination of C—H⋯O and C—H⋯π(arene) hydrogen bonds links the mol­ecules into sheets. When com­pound (II) was exposed to a strong acid in methanol, qu­anti­tative conversion occurred to give the ring-opened transesterification product methyl 2-[4-hy­droxy-1-(2-methyl­phen­yl)-1*H*-1,2,3-triazol-5-yl]benzoate, C_17_H_15_N_3_O_3_, (V), where the mol­ecules are linked by paired O—H⋯O hydrogen bonds to form centrosymmetric dimers.

## Abstract 2

An efficient synthesis of 1-aryl­isochromeno[3,4-*d*][1,2,3]triazol-5(1*H*)-ones is reported, along with the structures of four examples and the structure of a ring-opened transesterification product.

## Introduction   

Isocoumarins are an important building block in synthetic medicinal chemistry because they have shown inter­esting bio­activities, for example, as anti­coagulants (Oweida *et al.*, 1990 ▸), as herbicides (Zhang *et al.*, 2016 ▸) and as insecticides (Qadeer *et al.*, 2007 ▸). In order to gain access to com­pounds of this type in a straightforward way, a synthetic route has been developed using reactions between 2-formyl­benzoic acid, hydrogen cyanide and anilines to yield *N*-aryldi­amino­isocoumarins (Opatz & Ferenc, 2005 ▸). We have reported the structures of several com­pounds of this type (Vicentes *et al.*, 2013 ▸) and, more recently using such com­pounds as precursors, we have developed the synthesis of a new heterocyclic system, namely fused imidazolo­isocoumarins, as part of an exploration of possible synergies between the imidazole and isocoumarin pharmacophores (Rodríguez *et al.*, 2017 ▸).

The bioactivity of hybrid systems containing the 1,2,3-triazole unit has been reviewed recently (Xu *et al.*, 2019 ▸) and, with this in mind, we have now developed an efficient synthesis of 1-aryl­isochromeno[3,4-*d*][1,2,3]triazol-5(1*H*)-ones starting from the same *N*-aryldi­amino­isocoumarins as were used in the synthesis of imidazolo­isocoumarins (Rodríguez *et al.*, 2017 ▸). Thus, we now report the synthesis and spectroscopic characterization, and the mol­ecular and supra­molecular structures of four representative examples, namely, 1-phenyl­iso­chro­meno[3,4-*d*][1,2,3]triazol-5(1*H*)-one, (I), 1-(2-methyl­phen­yl)isochromeno[3,4-*d*][1,2,3]triazol-5(1*H*)-one, (II), 1-(3-chloro­phen­yl)isochromeno[3,4-*d*][1,2,3]triazol-5(1*H*)-one, (III), and 1-(4-chloro­phen­yl)isochromeno[3,4-*d*][1,2,3]triazol-5(1*H*)-one, (IV), carrying substituents at different positions in the pendent aryl group, along with those of a transesterification product, namely, methyl 2-[4-hy­droxy-1-(2-methyl­phen­yl)-1*H*-1,2,3-tri­azol-5-yl]benzoate, (V). Compounds (I)–(IV) were prepared by reaction of sodium nitrite in acetic acid with the corresponding 3-amino-4-aryl­amino-1*H*-isochromen-1-ones (**A**) (see Scheme 1); the precursors of type (**A**) having aryl = phenyl, 2-methyl­phenyl or 4-chloro­phenyl were prepared (Scheme 1) as reported previously (Rodríguez *et al.*, 2017 ▸), and the new analogue having aryl = 3-chloro­phenyl was prepared in the same way. The conversion of the precursors of type (**A**) to the products (I)–(IV) proceeds *via* the diazo­nium inter­mediate (**B**) (Scheme 1), which itself undergoes an intra­molecular cyclization to form the triazolo ring. It is important to stress here the necessity of using a weak acid, here acetic acid, in the diazo­tization of (**A**) to form (**B**), as isocoumarins often readily undergo ring opening in the presence of strong acids. To confirm this, a sample of com­pound (II) was stirred in methanol in the presence of aqueous hydro­chloric acid, resulting in a qu­anti­tative conversion of (II) to ester (V).

## Experimental   

### Synthesis and crystallization   

The known precursors of type (**A**) (see Scheme 1) having Ar = C_6_H_5_, 2-CH_3_C_6_H_4_ and 4-ClC_6_H_4_ were prepared as described previously (Rodríguez *et al.*, 2017 ▸); the new ana­logue having Ar = 3-ClC_6_H_4_ was prepared following the same procedure. Analytical data for 3-amino-4-(3-chloro­anilino)-1*H*-iso­chro­men-1-one: yellow solid, yield 71%, m.p. 451–452 K; IR (ATR, cm^−1^): 3456, 3319, 2922, 1701, 1592, 1474, 1306, 1089, 767, 679; NMR [CDCl_3_, the numbering of the chloro­phenyl ring follows that for com­pound (III)]: δ(^1^H) 8.15 (*dd*, *J* = 8.0, 0.7 Hz, 1H, H8), 7.53 (*t*, *J* = 7.6 Hz, 1H, H6), 7.20 (*t*, *J* = 7.6 Hz, 1H, H7), 7.16 (*d*, *J* = 8.1 Hz, 1H, H5), 7.10 (*t*, *J* = 8.0 Hz, 1H, H15), 6.76 (*dd*, *J* = 7.9, 1.1 Hz, 1H, H14), 6.65 (*t*, *J* = 2.0 Hz, 1H, H12), 6.56 (*dd*, *J* = 8.2, 1.6 Hz, 1H, H16), 4.85 (*s*, 1H, NH), 4.57 (*s*, 2H, NH_2_); δ(^13^C) 160.64 (CO), 154.78 (C3), 147.59 (C11), 140.40 (C4*A*), 135.65 (C13), 135.52 (C6), 130.86 (C15), 130.56 (C8), 124.23 (C7), 119.74 (C5), 119.29 (C14), 116.15 (C8A), 113.24 (C12), 111.59 (C16), 92.19 (C4); MS (EI, 70 eV): *m*/*z* (%) 285.9 (12) [*M*]^+^, 259.94 (31), 257.93 (100), 177.97 (16), 148.92 (20), 129.93 (21), 110.89 (17), 103.92 (17); HRMS (ESI–QTOF) found 287.0582, C_15_H_11_^35^ClN_2_O_2_ requires for [*M* + H]^+^ 287.0578.

For the synthesis of com­pounds (I)–(IV), sodium nitrite (153 mg, 2.22 mmol) was added to a suspension of the appropriate precursor (**A**) [1.09 mmol; 275 mg for (I), 290 mg for (II) and 313 mg for each of (III) and (IV)] in acetic acid (1.0 ml) and the resulting mixture was then stirred at ambient temperature for 5 min. The resulting solid precipitate was collected by filtration and washed with an aqueous solution of sodium hydrogen carbonate (10% *w*/*v*) and then with water. The crude solid products were purified by column chromatography on silica gel 60 (0.040–0.063 mm) using di­chloro­methane as eluent.
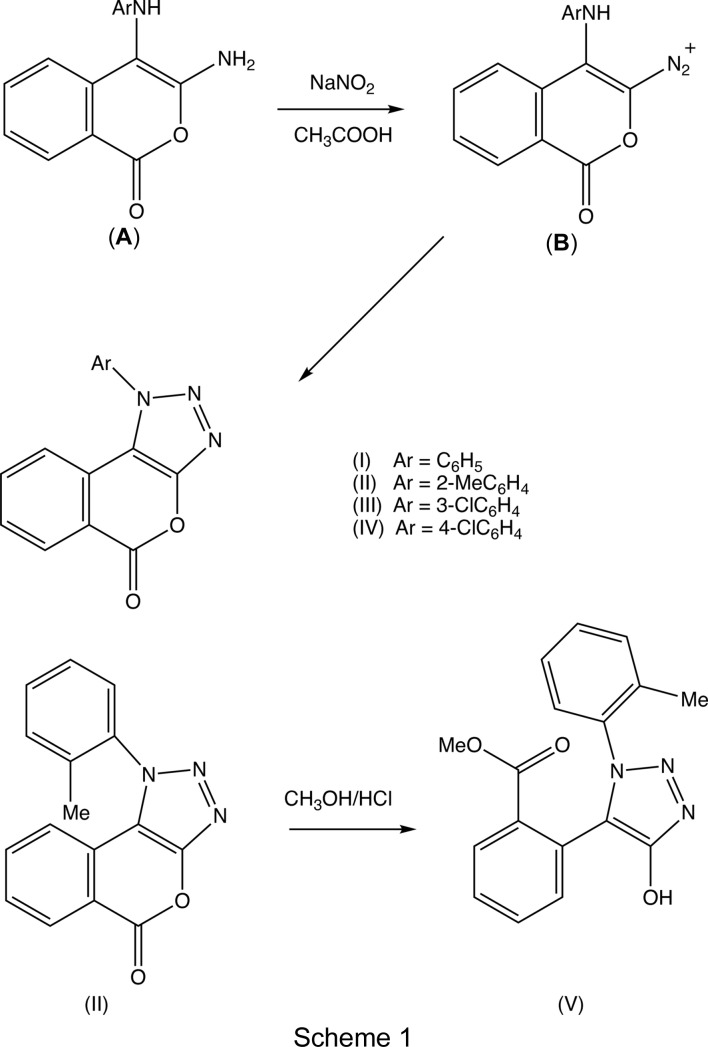


Analytical data for compound (I), colourless solid, yield 77%, m.p. 433–434 K; IR (ATR, cm^−1^): 3065, 1736, 1622, 1493, 1208, 1019, 763, 715; NMR (CDCl_3_): δ(^1^H) 8.46 (*dd*, *J* = 7.5, 1.6 Hz, 1H, H6), 7.72–7.55 (*m*, 7H, H7, H8, H12, H13, H14, H15, H16), 7.29 (*dd*, *J* = 7.8, 1.0 Hz, 1H, H9); δ(^13^C) 160.18 (CO), 154.65 (C3*A*), 136.89 (C11), 135.41 (C8), 132.84 (C6), 131.24 (C14), 130.23 (C13, C15), 129.79 (C7), 126.27 (C9*A*), 126.11 (C12, C16), 121.25 (C9), 120.57 (C9*B*), 115.26 (C5*A*); MS (EI, 70 eV): *m*/*z* (%) 262.98 (2) [*M*]^+^, 223.99 (35), 178.98 (100), 178.00 (29), 148.93 (36), 104.94 (23), 76.95 (35); HRMS (ESI–QTOF) found 264.0768, C_15_H_9_N_3_O_3_ requires for [*M* + H]^+^ 264.0768.

Analytical data for compound (II), colourless solid, yield 74%, m.p. 439–440 K; IR (ATR, cm^−1^): 1745, 1622, 1012, 987, 764, 680; NMR (CDCl_3_): δ(^1^H) 8.45 (*dd*, *J* = 7.1, 2.2 Hz, 1H, H8), 7.66–7.57 (*m*, 3H, H6, H7, H13), 7.55–7.48 (*m*, 2H, H14, H15), 7.46 (*dd*, *J* = 7.8, 1.6 Hz, 1H, H16), 6.92 (*dd*, *J* = 7.0, 2.1 Hz, 1H, H5), 2.10 (*s*, 3H, CH_3_); δ(^13^C) 160.22 (CO), 154.34 (C3*A*), 135.85 (C11), 135.69 (C8), 132.67 (C6), 131.89 (C14), 131.66 (C13), 129.78 (C7), 127.73 (C16), 127.38 (C15), 126.19 (C9*A*), 120.63 (C9), 120.46 (C9*B*), 115.62 (C5*A*), 17.32 (CH_3_); MS (EI, 70 eV): *m*/*z* (%) 276,97 (2) [*M*]^+^, 220.99 (16), 194.02 (15), 193.00 (100), 192.02 (21), 164.97 (22), 88.94 (22); HRMS (ESI–QTOF) found 278.0923, C_16_H_11_N_3_O_2_ requires for [*M* + H]^+^ 278.0924.

Analytical data for compound (III), yellow solid, yield 75%, m.p. 448–449 K; IR (ATR, cm^−1^): 3072, 2919, 2850, 1748, 1617, 1587, 1010, 885, 867, 783; NMR (CDCl_3_): δ(^1^H) 8.46 (*dd*, *J* = 7.9, 1.4 Hz, 1H, H6), 7.75–7.60 (*m*, 5H, H7, H8, H12, H14, H15), 7.56 (*ddd*, *J* = 7.7, 1.9, 1.4 Hz, 1H, H16), 7.34 (*dd*, *J* = 7.9, 0.6 Hz, 1H, H9); δ(^13^C) 159.94 (CO), 154.65 (C3*A*), 137.75 (C11), 136.03 (C13), 135.57 (C8), 132.95 (C6), 131.47 (C15), 131.22 (C14), 130.04 (C7), 126.46 (C12), 125.92 (C9*A*), 124.23 (C16), 121.20 (C9), 120.57 (C9*B*), 115.22 (C5*A*); MS (EI, 70 eV): *m*/*z* (%) 296.95 (2) [*M*]^+^, 214.96 (29), 213.98 (15), 212.01 (100), 177.98 (47), 150.97 (15), 74.94 (27); HRMS (ESI–QTOF) found 298.0380, C_15_H_8_^35^ClN_3_O_2_ requires for [*M* + H]^+^ 298.0378.

Analytical data for compound (IV), pink solid, yield 62%,, m.p. 490–492 K; IR (ATR, cm^−1^): 3087, 3066, 1740, 1620, 1457, 1217, 1013, 832, 764; NMR (CDCl_3_) δ(^1^H) 8.46 (*ddd*, *J* = 7.8, 1.5, 0.6 Hz, 1H, H6), 7.72–7.58 (*m*, 6H, H7, H8, H12, H13, H15, H16), 7.32 (*ddd*, *J* = 7.9, 1.3, 0.5 Hz, 1H, H9); δ(^13^C) 159.98 (CO), 154.69 (C3*A*), 137.45 (C11), 135.51 (C8), 135.30 (C14), 132.96 (C6), 130.53 (C13, C15), 130.00 (C7), 127.39 (C12, C16), 126.02 (C9*A*), 121.14 (C9), 120.59 (C9*B*), 115.25 (C5*A*); MS (EI, 70 eV): *m*/*z* (%) 296.93 (1.4) [*M*]^+^, 214.95 (31), 213.96 (15), 212.90 (100), 177.97 (39), 150.96 (14), 110.91 (13), 74.93 (25); HRMS (ESI–QTOF) found 298.0379, C_15_H_8_^35^ClN_3_O_2_ requires for [*M* + H]^+^ 298.0378.

For the conversion of com­pound (II) into com­pound (V), a sample of (II) (2.00 g, 7.22 mmol) and aqueous hydro­chloric acid (1 mol dm^−3^, 1 ml) were added to methanol (9 ml) and the resulting mixture was then stirred for 24 h at ambient temperature. The solvent was removed under reduced pressure and the resulting solid product was washed with an aqueous solution of sodium hydrogen carbonate (10% *w*/*v*) and then with water and finally dried in air to provide (V) as a colourless solid in qu­anti­tative yield (m.p. 463–464 K). Ana­lytical data: IR (ATR, cm^−1^): 2982, 2948, 1722, 1623, 1512, 1259, 764, 713; NMR (CDCl_3_): δ(^1^H) 10.48 (*s*, 1H, OH), 7.76 (*d*, *J* = 7.7 Hz, 1H, H3), 7.51 (*td*, *J* = 7.5, 1.2 Hz, 1H, H5), 7.44 (*t*, *J* = 7.2 Hz, 1H, H4), 7.40–7.29 (*m*, 2H, H213, H214), 7.26 (*td*, *J* = 7.7, 1.1 Hz, 1H, H215), 7.23–7.16 (*m*, 2H, H6, H216), 3.67 (*s*, 3H, OCH_3_), 2.02 (*s*, 3H, CH_3_); δ(^13^C) 166.53 (CO), 155.36 (C24), 135.61 (C211), 134.49 (C214), 131.80 (C5), 131.38 (C6), 131.09 (C213), 130.85 (C25), 129.94 (C3), 129.70 (C214), 128.85 (C4), 127.39 (C216), 126.52 (C215), 126.40 (C1), 119.03 (C2), 52.12 (OCH_3_), 17.07 (CH_3_); MS (EI, 70 eV): *m*/*z* (%) 309.00 (2) [*M*]^+^, 239.01 (16), 237.98 (100), 219.99 (21), 193.00 (70), 164.97 (23), 90.96 (30); HRMS (ESI–QTOF) found 310.1186, C_17_H_15_N_3_O_2_ requires for [*M* + H]^+^ 310.1186.

Crystals of com­pounds (I)–(V) suitable for single-crystal X-ray diffraction were grown by slow evaporation, at ambient temperature and in the presence of air, of solutions in chloro­form.

### Refinement   

Crystal data, data collection and structure refinement details are summarized in Table 1 ▸. All H atoms were located in difference maps. H atoms bonded to C atoms were subsequently treated as riding atoms in geometrically idealized positions, with C—H = 0.95 (alkenyl and aromatic) or 0.98 Å (CH_3_) and with *U*_iso_(H) = *kU*_eq_(C), where *k* = 1.5 for the methyl groups, which were allowed to rotate but not to tilt, and 1.2 for all other H atoms bonded to C atoms. For the H atom bonded to an O atom in com­pound (V), the atomic coordinates were refined with *U*_iso_(H) = 1.5*U*_eq_(O), giving an O—H distance of 0.90 (2) Å. Several low-angle reflections which had been attenuated by the beam stop were omitted, *i.e.* 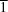
01 for (II) and 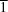
02 for (IV); in addition, one bad outlier reflection, *i.e.* 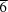
06, was omitted from the data set for (II) before the final refinements. For several of the refinements, the final analyses of variance showed unexpected values of *K* = [mean(*F*_o_^2^)/mean(*F*_c_^2^)] for the groups of the very weakest reflections. Thus, for (III) and (IV), respectively, −0.035 and −0.125 for 312 and 289 reflections in the *F*_c_/*F*_c_(max) ranges 0.000–0.008 and 0.000–0.010, and for (V), 3.550 for 339 reflections in the *F*_c_/*F*_c_(max) range 0.000–0.009; these values are probably statistical artefacts.

## Results and discussion   

**Figure 1:**
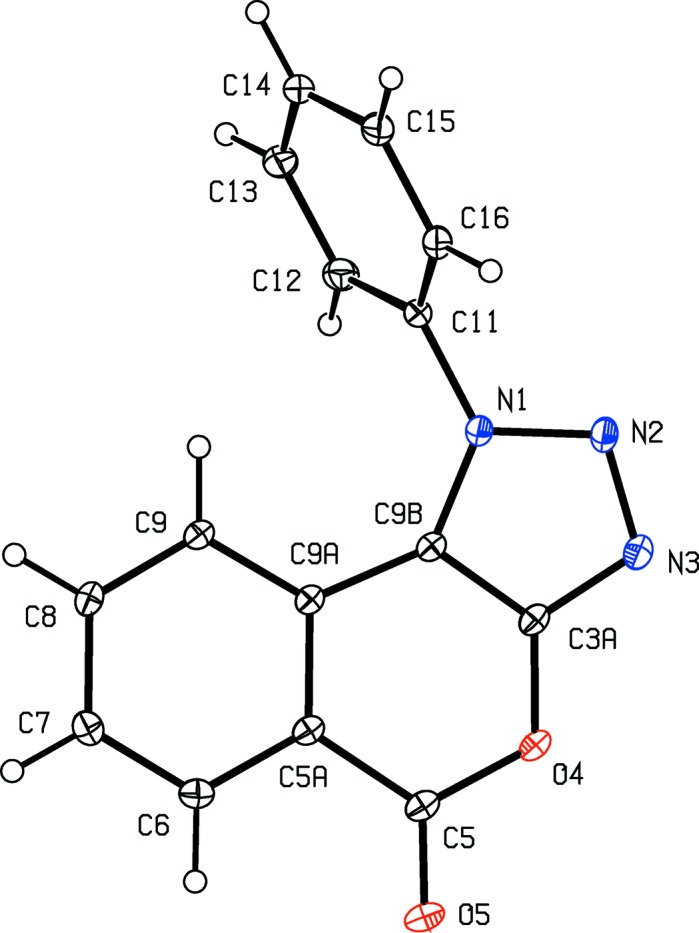
The mol­ecular structure of com­pound (I), showing the atom-labelling scheme. Displacement ellipsoids are drawn at the 30% probability level.

The constitutions of com­pounds (I)–(V) were all fully established by a combination of high-resolution mass spectrometry (HRMS), IR spectrosopy and ^1^H and ^13^C NMR spectroscopy, further confirmed by the structure analyses reported here (Figs. 1 ▸–5 ▸ ▸ ▸ ▸). The HRMS data for (I)–(IV) demonstrate the incorporation of an additional H atom, the IR data show the absence of an NH_2_ absorption around 3400 cm^−1^ and the ^1^H NMR spectra show the absence of signals around δ 4.5–5.0 arising from an amino group; these observations taken together confirm the conversion of the di­amino precursors of type (**A**) (Scheme 1) into the triazolo products (I)–(IV), whose constitutions were fully confirmed by the detailed assignments of the ^1^H and ^13^C NMR spectra (see §2.1). Hence, the constitutions of (I)–(IV) show clearly that the anti­cipated triazolo ring formation has occurred, with the additional N atom arising from the diazo­tization process; similarly, the con­stitution of (V) confirms the occurrence of a ring-opening transesterification process.

**Figure 2:**
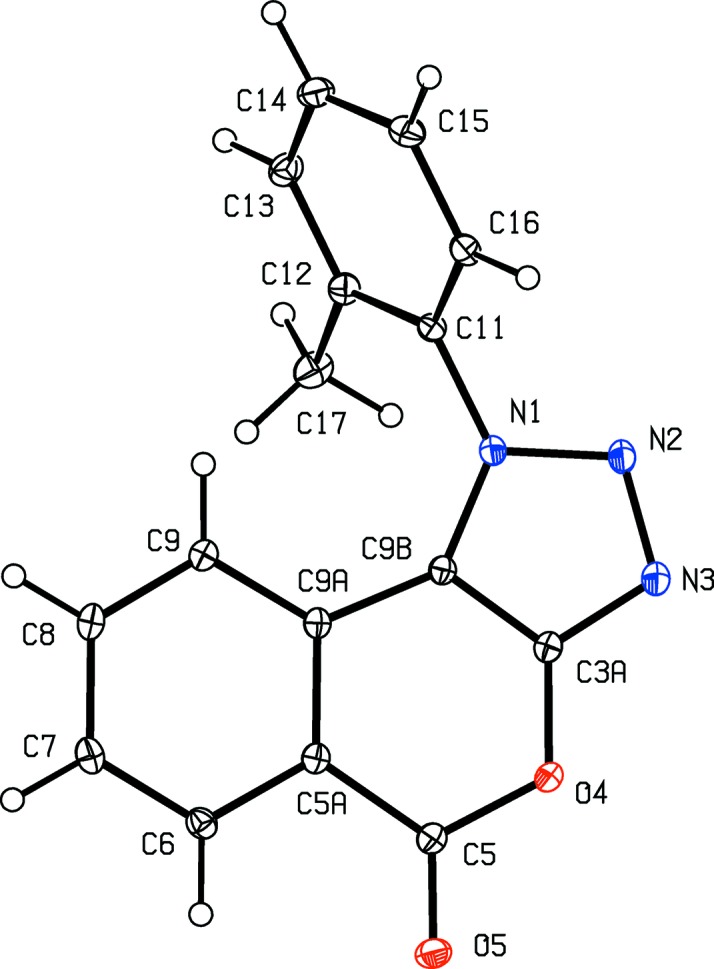
The mol­ecular structure of com­pound (II), showing the atom-labelling scheme. Displacement ellipsoids are drawn at the 30% probability level.

Aside from the orientation of the 2-methyl and 3-chloro substituents in com­pounds (II) and (III), respectively, the conformations of com­pounds (I)–(IV) are fairly similar; the dihedral angles between the triazolo ring and the pendent ring (C11–C16) are 65.32 (5), 64.59 (4), 45.48 (8) and 52.32 (9)° in (I)–(IV), respectively. The mol­ecules thus exhibit no inter­nal symmetry and so are conformationally chiral in the crystalline state; the centrosymmetric space groups (Table 1 ▸) confirm that (I)–(IV) have all crystallized as conformational racemates. For all of (I)–(IV), the reference mol­ecules were selected to have the same sign for the torsion angle N2—N1—C11—C12, or N2—N1—C11—C16 in the case of (III). A comparison of the conformation of ester (V) with that of its precursor (II) (Figs. 2 ▸ and 5 ▸) indicates that, in the crystalline state, there appear to have been rotations about both the bonds exocyclic to the triazolo ring in (V), along with a rotation about the bond linking the ester unit to the adjacent aryl ring. The significance of these differences is unclear. The bond lengths in (I)–(V) show no unusual features.

The supra­molecular assembly in com­pounds (I)–(IV) is dominated by contacts of C—H⋯N, C—H⋯O and C—H⋯π(arene) types (Table 2 ▸) and it is therefore worthwhile to specify the criteria under which such inter­actions are regarded here is structurally significant, or otherwise. Firstly, we discount all C—H⋯N and C—H⋯O contacts in which the *D*—H⋯*A* angle is less than 140°, as the inter­action energies associated with such contacts are likely to be extremely small (Wood *et al.*, 2009 ▸). Secondly, we discount all contacts involving methyl C—H bonds; these are not only of low acidity, but methyl groups CH_3_—*E* are generally undergoing very fast rotation about the C—*E* bonds, even in the solid state (Riddell & Rogerson, 1996 ▸, 1997 ▸). In particular, for methyl groups bonded to aryl rings, as found in (II) and (V), the rotation of the methyl group relative to the ring is subject to a sixfold rotation barrier, known to be in general extremely low, typically just a few J mol^−1^ rather than the more typical magnitude of a few kJ mol^−1^ (Tannenbaum *et al.*, 1956 ▸; Naylor & Wilson, 1957 ▸). Hence, there is just one significant inter­molecular C—H⋯*X* inter­action in each of (II), (III) and (V), involving atoms C13, C16 and O24, respectively, as the donors, and two each in (I) and (IV), involving as the donors C8 and C12 in (I), and C7 and C8 in (IV).

**Figure 6:**
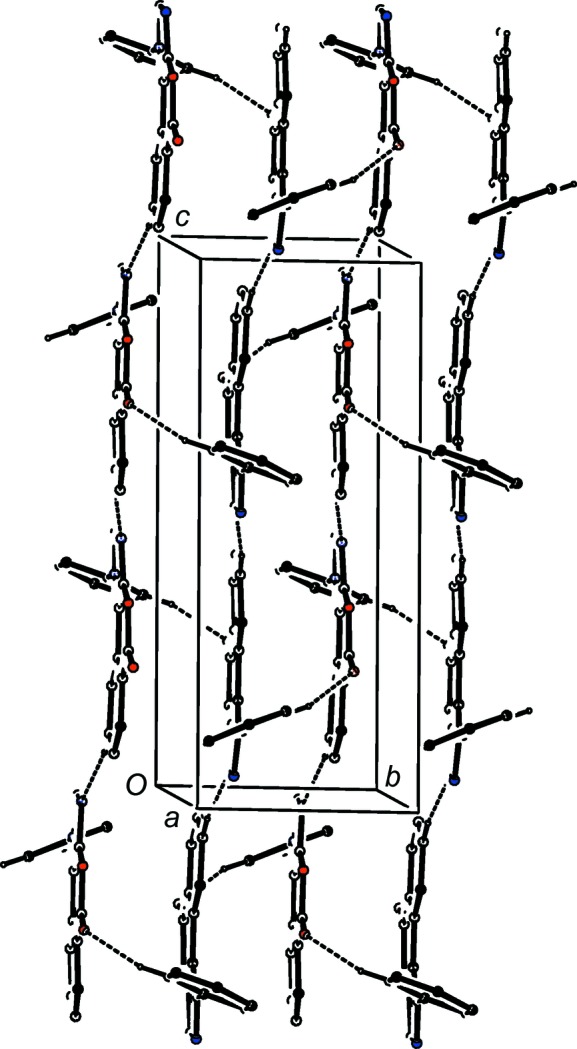
Part of the crystal structure of com­pound (I), showing the formation of a hydrogen-bonded sheet of 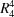
(28) rings parallel to (10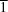). Hydrogen bonds are drawn as dashed lines and, for the sake of clarity, H atoms not involved in the motifs shown have been omitted.

The supra­molecular assembly in com­pound (I) is mediated by two hydrogen bonds, one each of the C—H⋯N and C—H⋯O types (Table 2 ▸). Mol­ecules which are related by an *n*-glide plane are linked by the C—H⋯N hydrogen bond to form a *C*(7) (Etter, 1990 ▸; Etter *et al.*, 1990 ▸; Bernstein *et al.*, 1995 ▸) chain running parallel to the [101] direction, while mol­ecules which are related by a 2_1_ screw axis are linked by a C—H⋯O hydrogen bond to form a *C*(9) chain running parallel to the [010] direction. The combination of these two chain motifs generates a sheet lying parallel to (10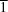) and built of 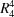
(28) rings (Fig. 6 ▸).

**Figure 7:**
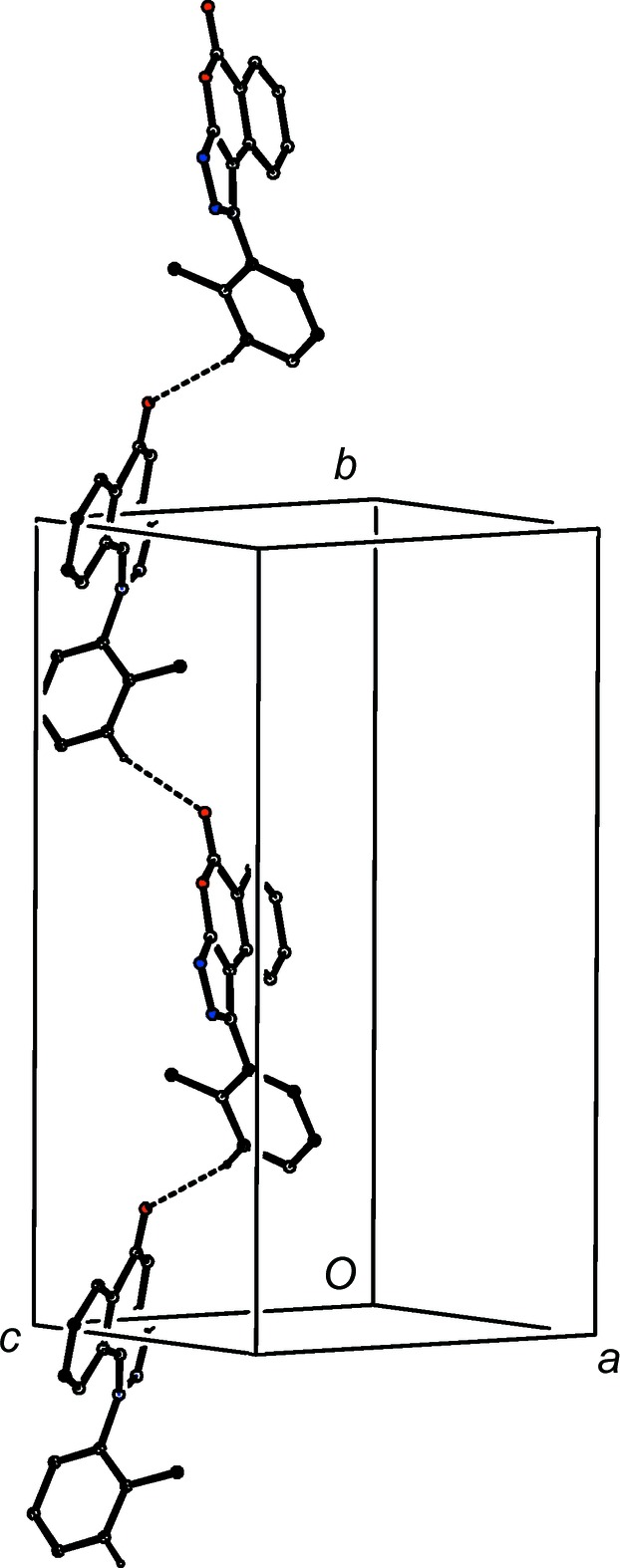
Part of the crystal structure of com­pound (II), showing the formation of a hydrogen-bonded *C*(10) chain parallel to [010]. Hydrogen bonds are drawn as dashed lines and, for the sake of clarity, H atoms not involved in the motifs shown have been omitted.

**Figure 8:**
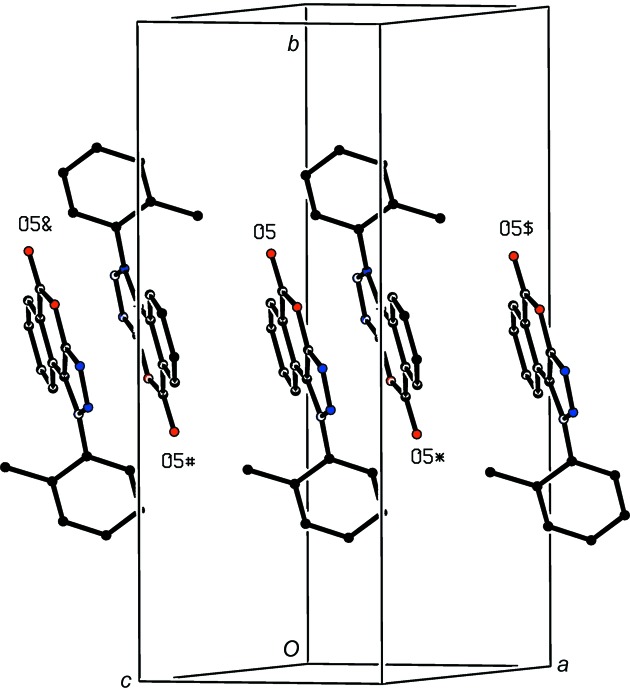
Part of the crystal structure of com­pound (II), showing the formation of a π-stacked chain parallel to [100]. For the sake of clarity, H atoms have all been omitted. O atoms marked with an asterisk (*), a hash (#), a dollar sign ($) or an ampersand (&) are at the symmetry positions (−*x* + 1, −*y* + 1, −*z* + 1), (−*x*, −*y* + 1, −*z* + 1), (*x* + 1, *y*, *z*) and (*x* − 1, *y*, *z*), respectively.

**Figure 9:**
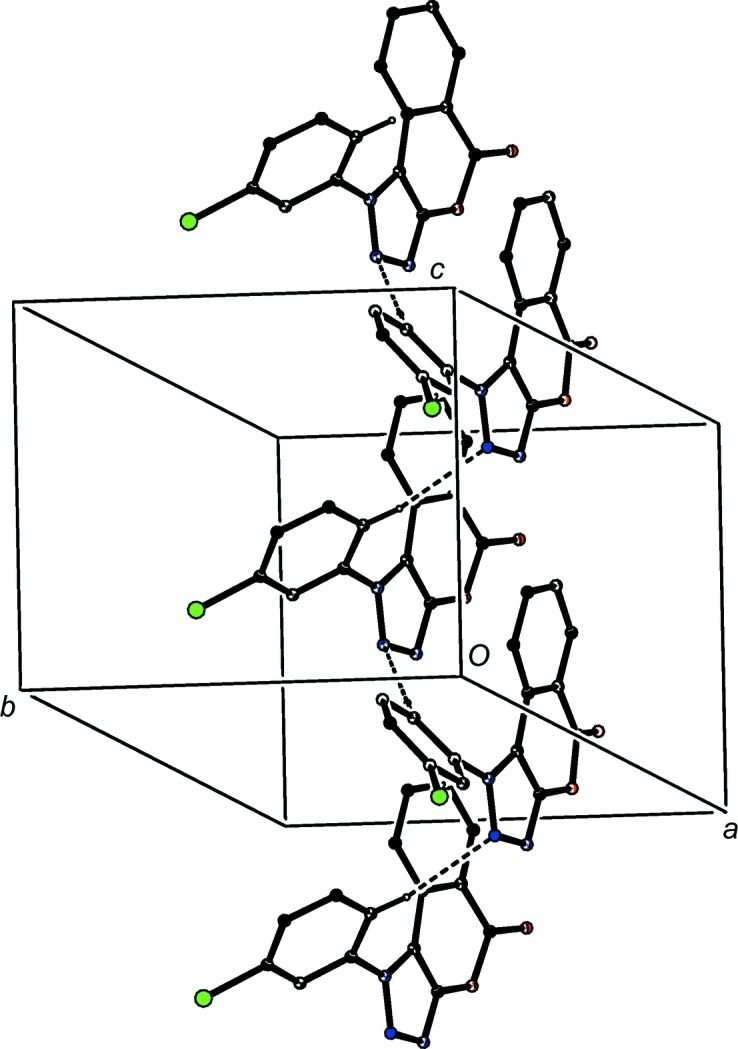
Part of the crystal structure of com­pound (III), showing the formation of a hydrogen-bonded *C*(5) chain parallel to [001]. Hydrogen bonds are drawn as dashed lines and, for the sake of clarity, H atoms not involved in the motif shown have been omitted.

For com­pound (II), a single C—H⋯O hydrogen bond links mol­ecules which are related by a 2_1_ screw axis to form a *C*(10) chain running parallel to the [010] direction (Fig. 7 ▸), and chains of this type are linked by two π–π stacking inter­actions, both involving the fused carbocyclic ring, which together generate a π-stacked chain running parallel to [100] (Fig. 8 ▸). The combination of these two motifs generates a sheet lying parallel to (001). There is again just one hydrogen bond in the structure of com­pound (III), this time of the C—H⋯N type, linking mol­ecules which are related by a *c*-glide plane to form a *C*(5) chain running parallel to the [001] direction (Fig. 9 ▸), but here there are no direction-specific inter­actions between adjacent chains.

**Figure 10:**
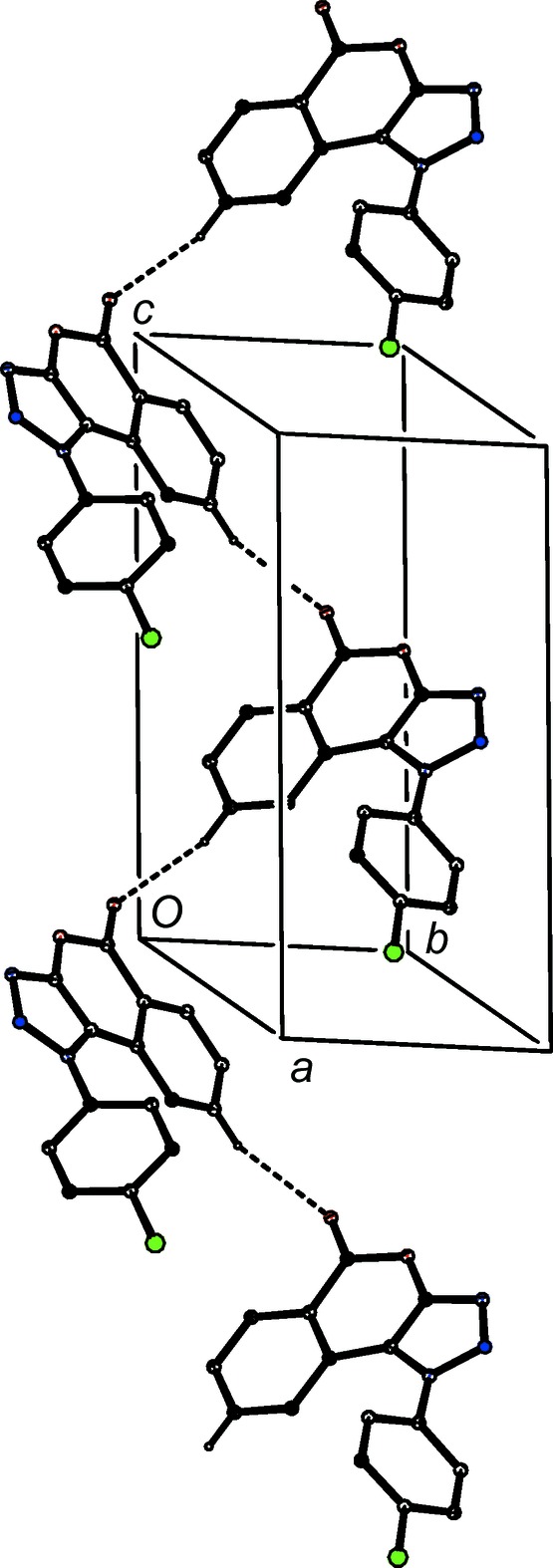
Part of the crystal structure of com­pound (IV), showing the formation of a hydrogen-bonded *C*(7) chain parallel to [001]. Hydrogen bonds are drawn as dashed lines and, for the sake of clarity, H atoms not involved in the motif shown have been omitted.

**Figure 11:**
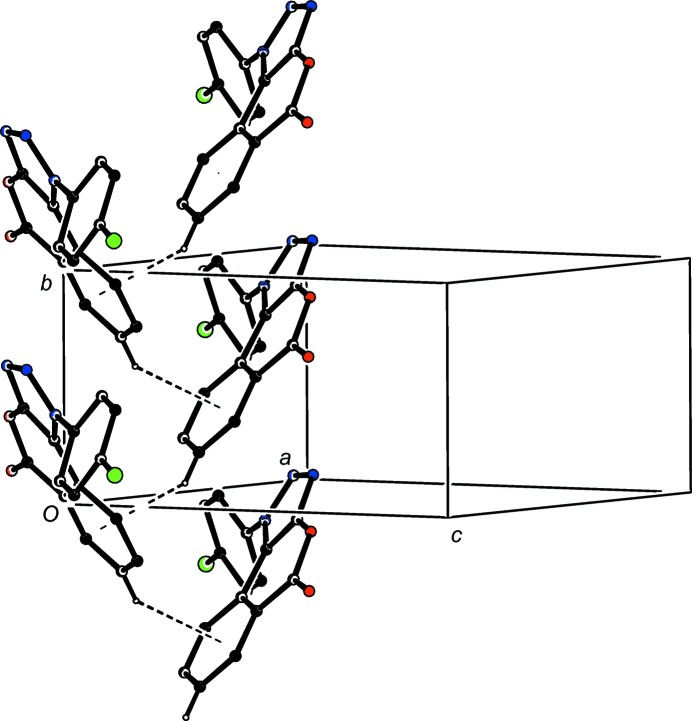
Part of the crystal structure of com­pound (IV), showing the formation of a hydrogen-bonded chain parallel to [010]. Hydrogen bonds are drawn as dashed lines and, for the sake of clarity, H atoms not involved in the motif shown have been omitted.

The assembly in com­pound (IV) is built from a combination of C—H⋯O and C—H⋯π(arene) hydrogen bonds (Table 2 ▸). The C—H⋯O hydrogen bond links mol­ecules which are related by a *c*-glide plane to form a *C*(7) chain running parallel to the [001] direction (Fig. 10 ▸). By contrast, mol­ecules which are related by a 2_1_ screw axis are linked by the C—H⋯π(arene) hydrogen bond to form a chain running parallel to the [010] direction (Fig. 11 ▸), and the combination of these two chain motifs generates a sheet lying parallel to (100).

**Figure 12:**
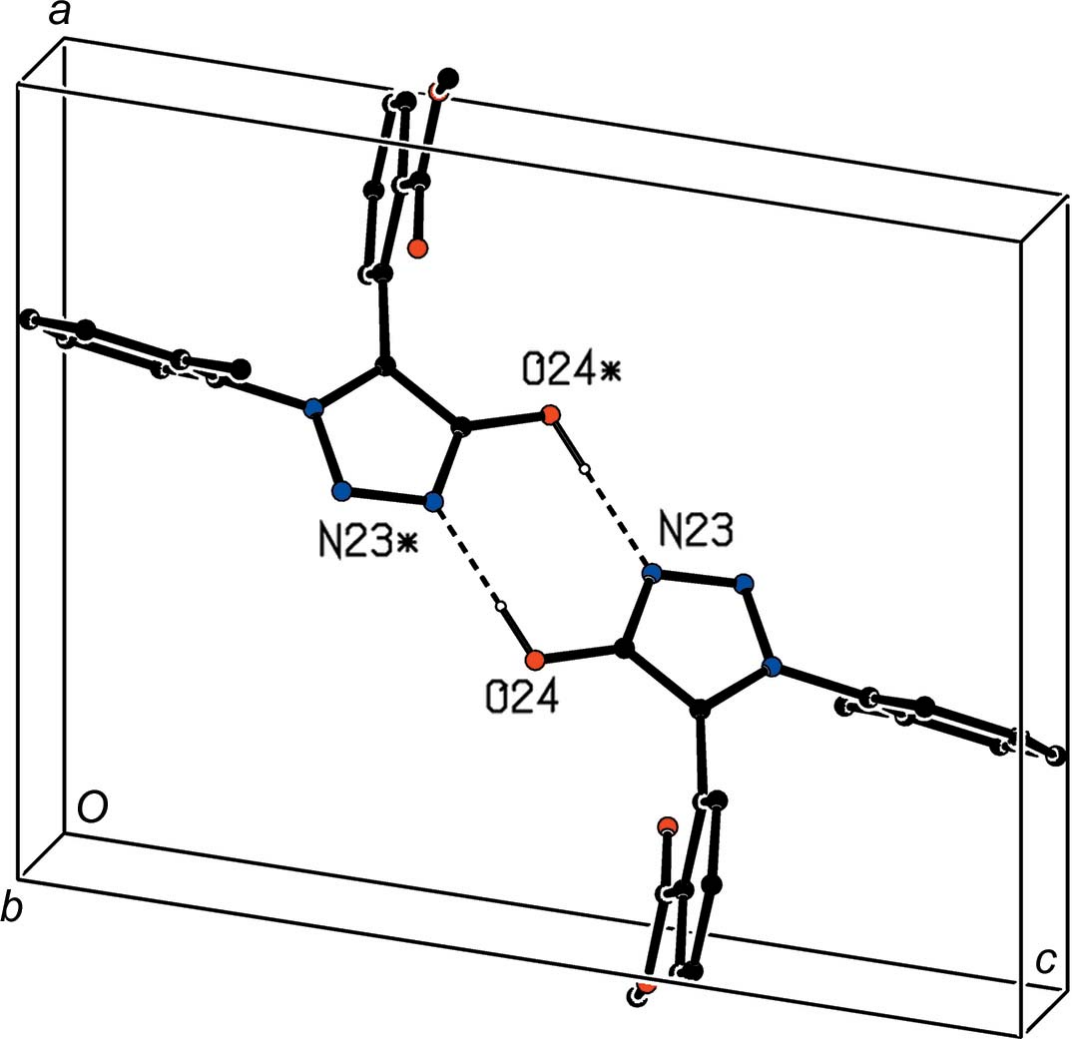
Part of the crystal structure of com­pound (V), showing the formation of a centrosymmetric 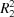
(8) dimer. Hydrogen bonds are drawn as dashed lines and, for the sake of clarity, H atoms bonded to C atoms have all been omitted. Atoms marked with an asterisk (*) are at the symmetry position (−*x* + 1, −*y* + 1, −*z* + 1).

Paired O—H⋯O hydrogen bonds link inversion-related pairs of mol­ecules of (V) to form a cyclic centrosymmetric 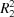
(8) dimer (Fig. 12 ▸), but there are no direction-specific inter­actions between adjacent dimer units.

Thus, minor variations in the substituent on the pendent aryl ring in com­pounds (I)–(IV) are associated with significant changes in the pattern of supra­molecular assembly. Whereas for the unsubstituted parent com­pound (I), the mol­ecules are linked into hydrogen-bonded sheets by a combination of C—H⋯N and C—H⋯O hydrogen bonds, the sheet formation in 4-chloro derivative (IV) is based on a combination of C—H⋯O and C—H⋯π(arene) hydrogen bonds. In each of the methyl com­pound (II) and the 3-chloro com­pound (III), a single hydrogen bond, of the C—H⋯O and C—H⋯N types, respectively, links the mol­ecules into simple chains; these chains form π-stacked sheets in (II), but not in (III).

In summary, therefore, we have developed a simple and efficient route to new 1-aryl­isochromeno[3,4-*d*][1,2,3]triazol-5(1*H*)-ones, with full spectroscopic and structural characterization of four examples, which show that small changes in substituents are associated with substantial changes in the patterns of supra­molecular aggregation, and we have demonstrated the necessity of using only a weak acid in the synthesis, along with the spectroscopic and structural characterization of a ring-opened derivative.

## Supplementary Material

Crystal structure: contains datablock(s) global, I, II, III, IV, V. DOI: 10.1107/S2053229620003757/sk3747sup1.cif

Structure factors: contains datablock(s) I. DOI: 10.1107/S2053229620003757/sk3747Isup2.hkl

Structure factors: contains datablock(s) II. DOI: 10.1107/S2053229620003757/sk3747IIsup3.hkl

Structure factors: contains datablock(s) III. DOI: 10.1107/S2053229620003757/sk3747IIIsup4.hkl

Structure factors: contains datablock(s) IV. DOI: 10.1107/S2053229620003757/sk3747IVsup5.hkl

Structure factors: contains datablock(s) V. DOI: 10.1107/S2053229620003757/sk3747Vsup6.hkl

Supporting information file. DOI: 10.1107/S2053229620003757/sk3747Isup7.cml

Supporting information file. DOI: 10.1107/S2053229620003757/sk3747IIsup8.cml

Supporting information file. DOI: 10.1107/S2053229620003757/sk3747IIIsup9.cml

Supporting information file. DOI: 10.1107/S2053229620003757/sk3747IVsup10.cml

Supporting information file. DOI: 10.1107/S2053229620003757/sk3747Vsup11.cml

CCDC references: 1990381, 1990380, 1990379, 1990378, 1990377

## Figures and Tables

**Figure 3:**
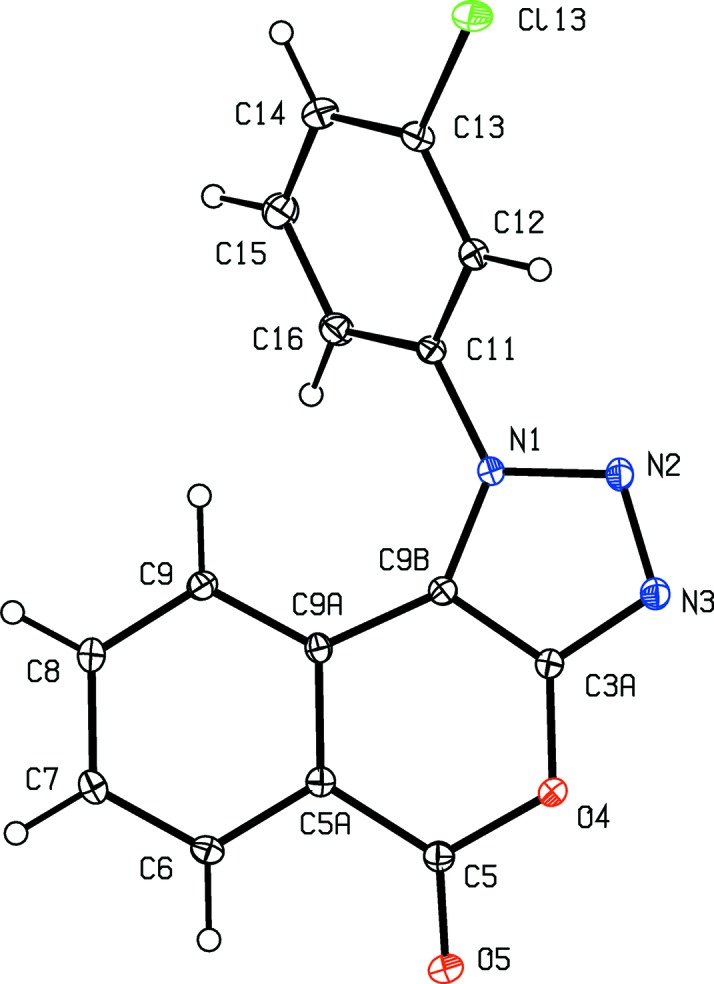
The mol­ecular structure of com­pound (III), showing the atom-labelling scheme. Displacement ellipsoids are drawn at the 30% probability level.

**Figure 4:**
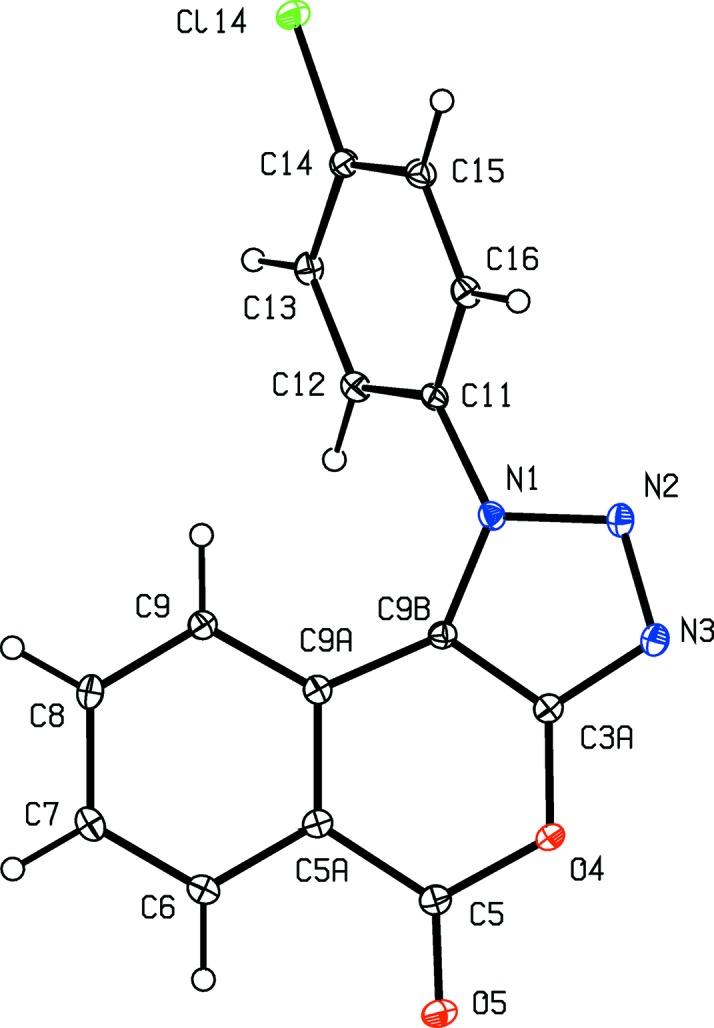
The mol­ecular structure of com­pound (IV), showing the atom-labelling scheme. Displacement ellipsoids are drawn at the 30% probability level.

**Figure 5:**
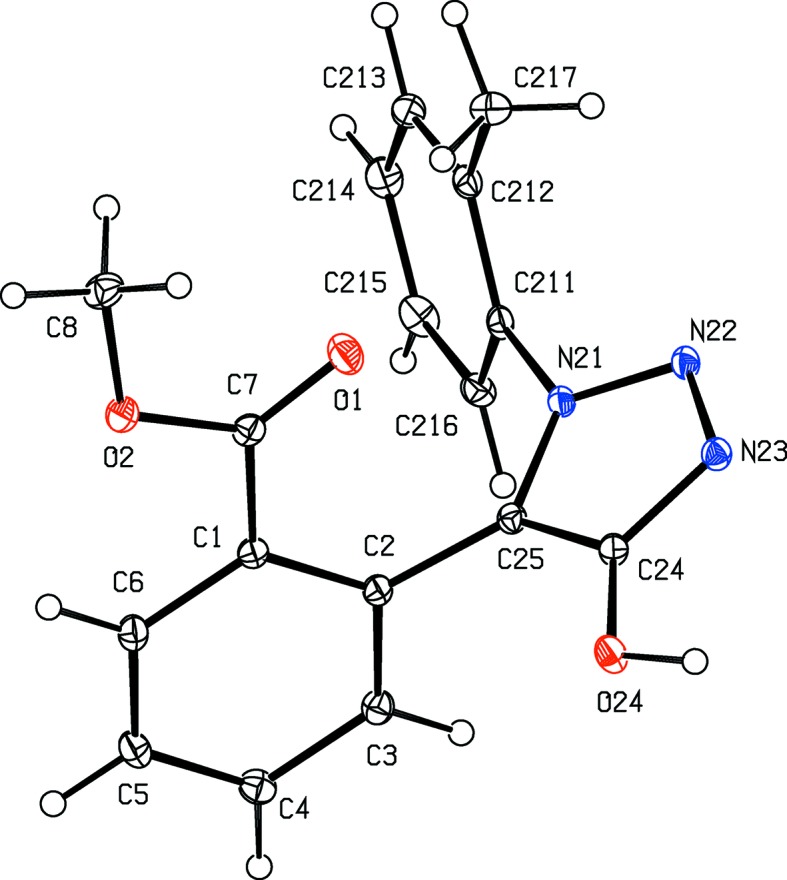
The mol­ecular structure of com­pound (V), showing the atom-labelling scheme. Displacement ellipsoids are drawn at the 30% probability level.

**Table:** For all structures: *Z* = 4. Experiments were carried out at 100 K with Mo *K*α radiation using a Bruker D8 Venture diffractometer. Absorption was corrected for by multi-scan methods (*SADABS*; Bruker, 2016 ▸).

	(I)	(II)	(III)
Crystal data			
Chemical formula	C_15_H_9_N_3_O_2_	C_16_H_11_N_3_O_2_	C_15_H_8_ClN_3_O_2_
*M*_r_	263.25	277.28	297.69
Crystal system, space group	Monoclinic, *P*2_1_/*n*	Monoclinic, *P*2_1_/*n*	Monoclinic, *P*2_1_/*c*
*a*, *b*, *c* (Å)	11.6814 (5), 6.4310 (3), 16.0589 (8)	7.5676 (5), 20.2663 (14), 9.2503 (7)	11.0993 (8), 12.8996 (9), 9.2234 (6)
β (°)	100.687 (2)	112.957 (3)	106.675 (2)
*V* (Å^3^)	1185.47 (10)	1306.33 (16)	1265.04 (15)
μ (mm^−1^)	0.10	0.10	0.31
Crystal size (mm)	0.20 × 0.12 × 0.06	0.28 × 0.17 × 0.16	0.20 × 0.12 × 0.05

Data collection			
*T*_min_, *T*_max_	0.936, 0.994	0.941, 0.985	0.895, 0.985
No. of measured, independent and observed [*I* > 2σ(*I*)] reflections	26237, 2747, 2400	36209, 3242, 2915	28194, 2905, 2495
*R*_int_	0.037	0.034	0.044
(sin θ/λ)_max_ (Å^−1^)	0.652	0.668	0.650

Refinement			
*R*[*F* ^2^ > 2σ(*F* ^2^)], *wR*(*F* ^2^), *S*	0.036, 0.103, 1.04	0.036, 0.098, 1.05	0.032, 0.089, 1.08
No. of reflections	2747	3242	2905
No. of parameters	181	191	190
H-atom treatment	H-atom parameters constrained	H-atom parameters constrained	H-atom parameters constrained
Δρ_max_, Δρ_min_ (e Å^−3^)	0.31, −0.21	0.31, −0.25	0.32, −0.36

**Table:** 

	(IV)	(V)
Crystal data		
Chemical formula	C_15_H_8_ClN_3_O_2_	C_17_H_15_N_3_O_3_
*M*_r_	297.69	309.32
Crystal system, space group	Monoclinic, *P*2_1_/*c*	Monoclinic, *P*2_1_/*c*
*a*, *b*, *c* (Å)	16.7331 (12), 5.9676 (4), 13.681 (1)	11.1518 (5), 9.3143 (4), 14.2417 (6)
β (°)	112.820 (3)	98.655 (2)
*V* (Å^3^)	1259.21 (16)	1462.46 (11)
μ (mm^−1^)	0.31	0.10
Crystal size (mm)	0.25 × 0.14 × 0.11	0.19 × 0.11 × 0.07

Data collection		
*T*_min_, *T*_max_	0.880, 0.967	0.960, 0.993
No. of measured, independent and observed [*I* > 2σ(*I*)] reflections	34775, 2891, 2413	33834, 3360, 3084
*R*_int_	0.056	0.036
(sin θ/λ)_max_ (Å^−1^)	0.650	0.650

Refinement		
*R*[*F* ^2^ > 2σ(*F* ^2^)], *wR*(*F* ^2^), *S*	0.034, 0.087, 1.12	0.042, 0.098, 1.07
No. of reflections	2891	3360
No. of parameters	190	213
H-atom treatment	H-atom parameters constrained	H atoms treated by a mixture of independent and constrained refinement
Δρ_max_, Δρ_min_ (e Å^−3^)	0.37, −0.26	0.34, −0.22

Computer programs: *APEX3* (Bruker, 2018 ▸), *SAINT* (Bruker, 2018 ▸), *SHELXT2014* (Sheldrick, 2015*a* ▸), *SHELXL2014* (Sheldrick, 2015*b* ▸) and *PLATON* (Spek, 2020 ▸).

**Table 2:** Hydrogen bonds and short inter­molecular contacts (Å, °) for com­pounds (I)–(V) *Cg*1 and *Cg*2 represent the centroids of the C5*A*/C6–C9/C9*A* and C1–C6 rings, respectively.

Compound	*D*—H⋯*A*	*D*—H	H⋯*A*	*D*⋯*A*	*D*—H⋯*A*
(I)	C8—H8⋯N3^i^	0.95	2.52	3.4641 (16)	173
	C12—H12⋯O5^ii^	0.95	2.50	3.4136 (16)	161
(II)	C7—H7⋯N3^iii^	0.95	2.55	3.2770 (15)	133
	C13—H13⋯O5^iv^	0.95	2.54	3.4083 (16)	151
	C15—H15⋯O5^v^	0.95	2.48	3.2482 (16)	138
(III)	C8—H8⋯N3^vi^	0.95	2.58	3.2465 (19)	127
	C16—H16⋯N2^vii^	0.95	2.56	3.500 (2)	169
(IV)	C6—H6⋯O5^viii^	0.95	2.53	3.287 (3)	137
	C8—H8⋯O5^ix^	0.95	2.57	3.578 (2)	161
	C15—H15⋯N3^x^	0.95	2.52	3.271 (2)	136
	C7—H7⋯*Cg*1^xi^	0.95	2.69	3.517 (2)	146
(V)	O24—H24⋯N23^xii^	0.90 (2)	1.77 (2)	2.6721 (15)	177.5 (19)
	C8—H8*A*⋯*Cg*2^xiii^	0.98	2.90	3.6605 (16)	135
	C8—H8*B*⋯*Cg*2^viii^	0.98	2.90	3.5007 (16)	120
	C8—H8*C*⋯O24^viii^	0.98	2.59	3.4004 (19)	140
	C215—H215⋯O24^xiv^	0.95	2.57	3.2972 (19)	134

Symmetry codes: (i) *x* + 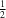, −*y* + 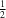, *z* + 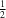; (ii) −*x* + 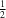, *y* + 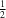, −*z* + 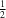; (iii) *x* − 1, *y*, *z* − 1; (iv) −*x* + 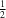, *y* − 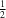, −*z* + 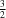; (v) −*x* + 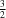, *y* − 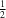, −*z* + 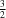; (vi) *x*, *y*, *z* + 1; (vii) *x*, −*y* + 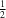, *z* + 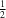; (viii) −*x*, −*y* + 1, −*z* + 1; (ix) *x*, −*y* + 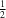, *z* − 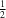; (x) *x*, −*y* + 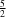, *z* − 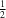; (xi) −*x*, *y* − 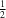, −*z* + 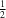; (xii) −*x* + 1, −*y* + 1, −*z* + 1; (xiii) −*x*, *y* − 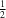, −*z* + 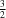; (xiv) *x*, −*y* + 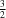, *z* + 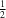.
